# Incidence of concurrent systemic injuries with traumatic proptosis and its effect on outcome – 100 dogs

**DOI:** 10.3389/fvets.2023.1271189

**Published:** 2024-01-16

**Authors:** Nicole Whinery, Rachel A. Allbaugh, Lionel Sebbag, Rebecca Walton

**Affiliations:** ^1^Emergency and Critical Care Department, VCA West Los Angeles Animal Hospital, Los Angeles, CA, United States; ^2^Department of Veterinary Clinical Sciences, Iowa State University, Ames, IA, United States

**Keywords:** ocular proptosis, trauma, temporary tarsorrhaphy, enucleation, general anesthesia

## Abstract

**Objective:**

To evaluate the incidence of concurrent systemic injuries in dogs with traumatic ocular proptosis and their effect on survival to discharge. Additionally, to evaluate for associations between the type of trauma, each presenting vital signs, minimum laboratory database findings including packed cell volume, total solids, plasma glucose and lactate concentrations, and the diagnosis of concurrent systemic injury and survival.

**Design:**

Retrospective study between the years 2017 and 2022.

**Setting:**

One university teaching hospital and one large, private practice.

**Animals:**

One hundred dogs presenting to the hospital with a diagnosis of traumatic ocular proptosis.

**Measurements and main results:**

Medical records were retrospectively reviewed; signalment, breed, sex, age, weight, date of presentation, type of trauma sustained, time from trauma to presentation, vitals on presentation, and minimum laboratory database findings including packed cell volume (PCV), total solids (TS), plasma glucose concentration, and plasma lactate concentration were recorded. A modified animal trauma triage (ATT) score was retrospectively calculated. A total of 17 dogs (17%) had concurrent systemic injury. Compared to dogs without systemic injuries, dogs with systemic injuries had a significantly lower body temperature [median 101.1F (38.3*C*) vs. 101.6F (38.6*C*); *P* = 0.008], significantly higher plasma glucose concentrations (125 mg/dL, 6.9 mmol/L vs. 112 mg/dL, 6.2 mmol/L; *P* = 0.012) and approaching statistical significance, lower PCV values (median 40 vs. 46%; *P* = 0.051).

**Conclusions:**

Dogs presenting with traumatic ocular proptosis do present with concurrent systemic, non-ocular injuries; however, these concurrent injuries do not seem to be associated with survival to discharge.

## Introduction

Ocular proptosis, or the sudden forward displacement of the globe out of the orbit with simultaneous entrapment of the eyelids behind the globe's equator, is a serious condition that most commonly occurs as a result of trauma (1–4). Following a patient's ocular and systemic assessment, rapid surgical intervention is required to minimize discomfort and damage to the eye, with two potential options including globe replacement with temporary tarsorrhaphy or globe removal via enucleation surgery (5). Decisions regarding globe replacement or removal depend on owner preference and factors affecting visual outcome such as cephalic conformation, pupillary light reflex, and number of extraocular muscles torn (1, 2, 6, 7). Both treatment options generally require general anesthesia and the risk associated with anesthesia may increase in patients with multiple traumatic injuries (8).

Concurrent damage to the globe or periocular tissue is common with proptosis and occurs in up to 69% of cases, including corneal ulceration, exposure keratitis and orbital fractures (2). On the other hand, concurrent systemic, non-ocular, injuries have seldom been reported in the veterinary literature, despite the traumatic etiology for developing ocular proptosis. Systemic injuries could affect patient stability and therefore increase risks of general anesthesia or negatively affect outcome including survival to discharge (9–11). Additionally, patients with severe concurrent systemic injuries may require delayed management of ocular proptosis, which may affect visual outcome (9–11). The main goal of this study was to evaluate the incidence of concurrent systemic injuries in dogs with traumatic ocular proptosis and to assess their effect on survival to discharge. A secondary study objective was to evaluate for potential associations among the type of trauma, presenting vital signs, minimum laboratory database findings, and the diagnosis of concurrent systemic injury and survival to discharge. We hypothesized that the majority of dogs with ocular proptosis would have additional concurrent systemic injuries due to the trauma event and the diagnosis of concurrent injuries would be associated with survival to discharge.

## Materials and methods

### Data collection

The online medical record databases of a University Teaching Hospital and large private practice hospital were retrospectively reviewed for dogs diagnosed with globe proptosis between the years 2017 and 2022. Inclusion criteria included dogs presenting with ocular proptosis secondary to trauma. Cases were excluded if the proptosis was secondary to any non-traumatic etiology or if the medical record was incomplete. For each record, the following admission data were collected: breed, sex, age, weight, date of presentation, type of trauma sustained, time from trauma to presentation, vitals on presentation including temperature, heart rate and respiratory rate, and minimum laboratory database including packed cell volume (PCV), total solids (TS), plasma glucose concentration, and plasma lactate concentration. Packed cell volume was obtained by collecting the blood sample and placing it in microhematocrit tubes, which were spun in a micro hematocrit centrifuge and obtained via a microhematocrit reader. Total solids were obtained by placing a drop of serum on a refractometer and assessing the concentration of the plasma. Plasma glucose and plasma lactate concentrations were obtained via blood gas analyzer^1^ or bedside glucometer^2^ and lactometer.^3^ Additional data collected included concurrent systemic injury including traumatic brain injury, thoracic injury, abdominal injuries, or orthopedic injuries, as well as survival to discharge and visual retention. Traumatic brain injury was diagnosed based on physical and neurologic exam, thoracic and orthopedic injuries were diagnosed based on radiographic evaluation and abdominal injuries were based on focused assessment with point-of-care ultrasound. A modified animal trauma triage (ATT) score was retrospectively calculated by a single evaluator.

### Data analysis

Each dog was classified as “survivor” or “non-survivor” if they did or did not survive to discharge, respectively. Further, each dog was classified as “systemically injured” or “non-systemically injured” based on any description of concurrent systemic injury other than proptosis in the medical records. Statistical comparisons between groups (survivor vs. non-survivor or systemically injured vs. non-systemically injured) was performed using Mann-Whitney rank sum tests for numerical data and Chi-square tests for categorical data. Statistical analyses were performed with SigmaPlot 14.5 (Systat software, Point Richmond, CA), and *P*-values < 0.05 were considered significant.

## Results

A total of 136 cases were identified in the electronic database. One hundred dogs met the inclusion criteria while 36 cases were excluded due to trauma not being noted as the primary etiology of proptosis (*n* = 25) or incomplete medical records (*n* = 11). Thirty-eight dogs were castrated males, 26 were spayed female, 23 were intact females, and 13 were intact males. The most commonly reported breeds were Chihuahua (20), Shih tzu (19), mixed breed dogs (17), Yorkshire terrier (6), Terrier (5), Pug (4), Australian Shepherd (4), French Bulldog (3), Pomeranian (3), Maltese (3), Poodle (3), Pekingese (2), and Miniature Pincher (2). The following breeds were represented once: Beagle, German Shorthair Pointer, Blue Heeler, Boston Terrier, Papillion, Cocker Spaniel, German Shepherd Dog, Brussels Griffon, and Boxer. The median age was 7 years [interquartile range (IQR) 4–9 years]. The median body weight was 5.46 kg (IQR 4.1–8.15 kg). Seventy-seven dogs had traumatic proptosis secondary to bite wounds, 15 due to blunt trauma, three secondary to choking, one after falling from a height and 4 were unwitnessed but suspected trauma based on the environment they were found in.

The median time from trauma or identification of the proptosis to presentation was 2 h (IQR 1–5 h). Presenting vital parameters and minimum laboratory data are presented in Table 1. Modified ATT scores were retrospectively calculated. A score was able to be calculated for 95 dogs with a median (IQR) score of 0 (0–1). The median (IQR) modified ATT score in dogs without concurrent systemic injuries was 0 (0–1) and 1 (0–2) in dogs with concurrent systemic injuries.

**Table 1:** Presenting vital signs including temperature, heart rate, and respiratory rate.

	**Total number**	**Median (IQR)**	**Reference range:**
Temperature	89	101.6F, 38.7*C* (100.8–102.3F, 38.2–39*C*)	99.5–102.5F, 37.5–39.2*C*
Heart rate	97	130 bpm (110–150 bpm)	70–120 bpm
Respiratory rate	94	36 bpm (30–40 bpm)	15–30 bpm
Packed cell volume	53	45% (40–50%)	35–50%
Total solids	53	7.2 g/dL (6.28–7.8 g/dL)	5.4–7.5 g/dL
Plasma glucose	57	114 g/dL, 6.3 mmol/L (105–127 g/dL, 5.8–7.1 mmol/L)	80–120 g/dL, 4.4–6.6 mmol/L
Plasma lactate	45	2.15 mmol/L (1.8–3.05 mmol/L)	< 2.5 mmol/L

Presenting minimum laboratory data base. Median and (IQR) reported.

A total of 17 dogs (17%) had concurrent systemic injury including. Eight dogs (8%) had evidence of traumatic brain injury based on presenting neurologic exam. One dog (1%) with blunt force trauma had evidence of thoracic injury based on radiographs, diagnosed with pulmonary contusions. One dog had a hemoabdomen (1%). Ten dogs (10%) had skin lacerations noted, including the head (7), neck (3) and thorax (1), 5 (5%) dogs had skull fractures including mandibular (3), orbit (1) and zygomatic bone (1) and 1 dog (1%) had a spinal fracture.

### Systemically injured vs. non-systemically injured groups

Compared to dogs without systemic injuries, dogs with systemic injuries had a significantly lower body temperature [median 101.1F (38.3*C*) vs. 101.6F (38.6*C*); *P* = 0.008], significantly higher plasma glucose concentrations (125 mg/dL, 6.9 mmol/L vs. 112 mg/dL, 6.2 mmol/L; *P* = 0.012) and approaching statistical significance, lower PCV values (median 40 vs. 46%; *P* = 0.051).

### Survivor vs. non-survivors

Ninety-seven dogs (97%) survived to discharge. Fourteen of the 17 dogs with concurrent systemic injury survived to discharge (82%). One of the dogs that did not survive to discharge had concurrent systemic injuries. This dog that did not survive to discharge had a hemoabdomen and spinal fracture due to blunt trauma and was euthanized. Two dogs that did not survive to discharge did not have concurrent systemic injuries noted. One of the dogs experienced cardiopulmonary arrest with suspected upper airway obstruction. This dog died ~12 h following general anesthesia and enucleation, necropsy was consistent with mitral valve and tricuspid valve endocardiosis and no other signs of trauma noted. The other dog died during anesthetic induction. This dog became apneic during anesthesia induction and experienced cardiopulmonary arrest and return of spontaneous circulation was not achieved.

There was no statistical difference in time to presentation (*P* = 0.888), temperature on presentation (*P* = 0.712), heart rate (*P* = 0.372), or respiratory rate (*P* = 0.949) between animals that did and did not survive to discharge. There was no statistical difference in PCV (*P* = 0.179), TS (*P* = 0.442), plasma glucose concentration (*P* = 0.299), or plasma lactate concentration (*P* = 0.090) in animals that did and did not survive to discharge.

In this study, 44 dogs had a temporary tarsorrhaphy performed while 54 dogs had an enucleation performed. No patient required conversion from attempted tarsorrhaphy to enucleation. One dog was euthanized prior to treatment and one dog died prior to treatment for the ocular proptosis. Of the 44 dogs that had a temporary tarsorrhaphy performed 11 had concurrent systemic injuries. Seven of the dogs with concurrent systemic injuries had vision assessed and two of those dogs were visual. There was no difference in visual retention in systemically injured vs. non-systemically injured animals (*P* = 0.218).

## Discussion

In this study, a small number of dogs (17%) had concurrent non-ocular injuries when presenting for evaluation of traumatic ocular proptosis. In contrast with our original hypothesis, the presence of a concurrent systemic injury did not affect survival to discharge in dogs that presented with an ocular proptosis. One dog with concurrent systemic injury did not survive to discharge and was euthanized. This dog had significant concurrent injuries including spinal fracture and hemoabdomen. Two dogs (2%) without concurrent systemic injuries died. The lack of correlation between concurrent systemic injuries and survival to discharge may be due to the low number of animals that did not survive to discharge. Additionally, the lack of correlation may have resulted in fact that the majority of concurrent systemic injuries that were diagnosed in this study population were mild, based on the low modified ATT scores in all groups, and therefore did not affect survival to discharge.

As a secondary objective, the present study evaluated for potential associations between presenting vital signs and minimum laboratory database findings at the time of presentation with the presence of concurrent systemic injuries and survival to discharge. In this study, dogs presenting with concurrent systemic injuries had significantly lower body temperatures than those without concurrent systemic injury. Dogs with concurrent systemic injuries had lower body temperatures, which may be due to shock resulting in hypothermia. It is important to note that while statistically significant, both groups still had temperatures within reference range.

Minimum laboratory database findings such as plasma glucose concentration and plasma lactate concentration have been associated with the severity of injury and non-survival rates in previous veterinary studies (12–14). Further, plasma glucose concentrations were significantly higher in patients with concurrent systemic injury *vs*. ones without concurrent systemic injury. This hyperglycemia may be due to increased levels of circulating catecholamines and cortisol, which has been noted with traumatic brain injury and other trauma (12, 15–17). Here, dogs with concurrent systemic injuries had lower PCV values, approaching statistical significance, than patients without concurrent injuries, which may be attributed to mild hemorrhage at the site of ocular proptosis or at additional locations of trauma. It is important to note in this study, while approaching statistical significance, differences in PCV between systemically injured and non-systemically injured, the median values for both groups were still within reference ranges.

While minor differences were noted between PCV and plasma glucose concentrations between patients with concurrent systemic injuries and those without, no point-of-care diagnostics were associated with survival to discharge. This finding is similar to previous studies of blunt trauma that have noted no difference in PCV and plasma glucose concentrations between survivors and non-survivors (18).

The main limitation is the retrospective nature of the work. Not all cases had point-of-care diagnostics performed at the time of presentation. Additionally, the majority of patients did not have additional diagnostic testing to evaluate for all potential concurrent systemic injuries (e.g., thoracic or abdominal imaging) beyond a physical exam, which may have resulted in underdiagnosis of concurrent systemic injuries. Another limitation in this study is that traumatic ocular proptosis has an excellent prognosis and only three dogs did not survive to discharge, therefore, it was difficult to evaluate for an association between survival to discharge and concurrent systemic injuries.

In conclusion, dogs presenting with traumatic ocular proptosis may have concurrent non-ocular injuries, therefore thorough patient assessment is recommended. While concurrent systemic injuries did not affect survival to discharge, these injuries may affect patient comfort, patient management, and risks of general anesthesia. Two dogs that did not have any evidence of concurrent systemic injuries died, therefore anesthetic and general risks should be clearly discussed with owners in all cases of traumatic ocular proptosis and overnight hospitalization for post-operative monitoring may be warranted.

## Data availability statement

The raw data supporting the conclusions of this article will be made available by the authors, without undue reservation.

## Author contributions

NW: Conceptualization, Data curation, Methodology, Writing – original draft, Writing – review & editing. RA: Conceptualization, Writing – review & editing, Supervision. LS: Writing – review & editing, Formal analysis, Methodology. RW: Methodology, Writing – review & editing, Conceptualization, Data curation, Supervision, Writing – original draft.

## Footnotes

^1^Stat Profile Prime—NOVA biomedical, MA, USA.

^2^AlphaTrak—Zoetis, NJ, USA.

^3^LactatePlus—Nova Biomedical, MA, USA.
